# Supplementary material for: Lower synaptic density is associated with depression severity and network alterations
Source: Nat Commun. 2019 Apr 4;10:1529. doi: 10.1038/s41467-019-09562-7 (PMC6449365; doi:10.1038/s41467-019-09562-7)
Supplement: Supplementary file 1 — Supplementary Information [file 41467_2019_9562_MOESM1_ESM.pdf]

Supplementary Information

Lower synaptic density is associated with depression severity and network alterations

Holmes et al.

Supplementary Tables

Supplementary Table 1. V_T values (partial volume corrected; PVC) across groups

Region	HC group (n=21)	Low severity group (n=14)	High severity group (n=12)	Low severity vs. HC			High severity vs. HC		
				p-value	% diff	Cohen's d	*p*-value	% diff	Cohen's d
dlPFC	37.45 (4.01)	38.67 (3.97)	31.86 (5.67)	0.386	3.23	0.30	0.002	14.94	1.14
ACC	31.55 (3.34)	32.48 (3.18)	26.58 (4.27)	0.419	2.94	0.29	0.001	15.76	1.30
Hippocampus	19.11 (2.11)	19.12 (1.54)	16.22 (3.06)	0.990	0.04	0.01	0.003	15.14	1.10
Cerebellum	22.35 (1.99)	22.96 (3.37)	19.19 (2.12)	0.478	2.43	0.22	<0.001	14.17	1.54
Frontal cx	34.22 (3.75)	35.49 (3.72)	28.99 (4.99)	0.332	3.71	0.34	0.002	15.28	1.18
Occipital cx	34.89 (3.89)	35.80 (4.44)	29.98 (4.85)	0.527	2.60	0.22	0.003	14.08	1.12
Parietal cx	36.91 (4.58)	38.64 (4.71)	31.50 (5.82)	0.288	4.68	0.37	0.006	14.66	1.03
Putamen	29.07 (2.84)	30.58 (3.15)	25.10 (3.83)	0.150	5.18	0.50	0.002	13.67	1.18
Temporal cx	34.24 (3.48)	34.94 (3.95)	29.32 (4.56)	0.588	2.03	0.19	0.001	14.38	1.21
Thalamus	19.95 (2.45)	20.33 (3.04)	18.72 (3.53)	0.689	1.88	0.14	0.245	6.18	0.40

Values are presented as mean (SD)

Supplementary Table 2. V_T/f_p values across groups

Region	HC group (n=21)	Low severity group (n=14)	High severity group (n=12)	Low severity vs. HC			High severity vs. HC		
				p-value	% diff	Cohen's d	*p*-value	% diff	Cohen's d
dlPFC	134.61 (15.48)	140.38 (14.33)	116.21 (22.15)	0.274	4.29	0.39	0.009	13.67	0.98
ACC	113.28 (11.91)	117.98 (12.26)	96.74 (15.45)	0.266	4.15	0.40	0.002	14.60	1.18
Hippocampus	68.79 (9.22)	69.44 (5.270)	58.90 (9.86)	0.818	0.93	0.09	0.007	14.38	1.09
Cerebellum	76.15 (19.75)	83.26 (11.14)	69.96 (9.17)	0.231	9.34	0.43	0.315	8.14	0.39
Frontal cx	122.92 (13.86)	128.86 (13.49)	105.72 (19.55)	0.218	4.83	0.43	0.006	13.99	1.05
Occipital cx	125.54 (16.37)	129.77 (13.77)	109.33 (19.21)	0.431	3.37	0.21	0.015	12.91	0.97
Parietal cx	132.52 (16.66)	140.25 (16.81)	114.77 (22.27)	0.190	5.83	0.41	0.014	13.40	0.97
Putamen	104.46 (10.93)	110.94 (10.54)	91.65 (16.25)	0.091	6.20	0.55	0.011	0.60	0.95
Temporal cx	122.98 (12.62)	126.69 (12.51)	106.74 (17.02)	0.399	3.02	0.31	0.004	13.20	1.08
Thalamus	71.45 (12.62)	73.79 (10.83)	68.22 (13.24)	0.436	3.27	0.20	0.359	4.52	0.26

Values are presented as mean (SD)

Supplementary Table 3. V_T values (without PVC) across groups

Region	HC group (n=21)	Low severity group (n=14)	High severity group (n=12)	Low severity vs. HC			High severity vs. HC		
				p-value	% diff	Cohen's d	*p*-value	% diff	Cohen's d
dlPFC	23.64 (3.06)	25.08 (2.71)	20.85 (3.41)	0.164	6.09	0.49	0.022	11.78	0.86
ACC	21.89 (2.76)	23.02 (1.89)	19.30 (3.02)	0.195	5.12	0.48	0.018	11.85	0.90
Hippocampus	14.86 (1.65)	14.99 (1.28)	12.88 (2.34)	0.806	0.87	0.09	0.008	13.33	0.99
Cerebellum	15.54 (1.93)	16.51 (2.43)	14.01 (1.50)	0.202	6.18	0.44	0.024	9.85	0.90
Frontal cx	22.00 (2.80)	23.28 (2.28)	19.24 (3.00)	0.164	5.82	0.50	0.012	12.54	0.95
Occipital cx	21.94 (2.94)	23.10 (2.28)	19.37 (2.87)	0.222	5.30	0.44	0.021	11.70	0.88
Parietal cx	22.27 (3.00)	23.90 (2.38)	19.66 (3.19)	0.098	7.30	0.61	0.025	11.74	0.85
Putamen	23.61 (2.44)	24.02 (2.28)	20.14 (3.08)	0.634	1.82	0.18	0.001	14.70	1.26
Temporal cx	23.08 (2.79)	23.98 (2.44)	20.34 (2.87)	0.333	3.90	0.34	0.012	11.85	0.97
Thalamus	14.59 (2.06)	15.16 (1.36)	13.25 (2.46)	0.363	3.96	0.32	0.106	9.13	0.59

Values are presented as mean (SD)

Supplementary Table 4. Parent fraction and metabolite-corrected input function values across groups

	HC group (n=21)	Low severity group (n=14)	High severity group (n=12)	HC vs. low severity *p*-value	HC vs. high severity *p*-value
Parent fraction 30 min (%)	28 (7)	23 (7)	27 (8)	0.08	0.81
Parent fraction 60 min (%)	25 (5)	22 (6)	23 (5)	0.14	0.39
Metabolite-corrected input function 40-60 min (SUV)	0.25 (0.05)	0.22 (0.05)	0.24 (0.06)	0.12	0.27

Values are presented as mean (SD)

Supplementary Figure 1. No correlation between injected mass dose and V_T across primary ROIs

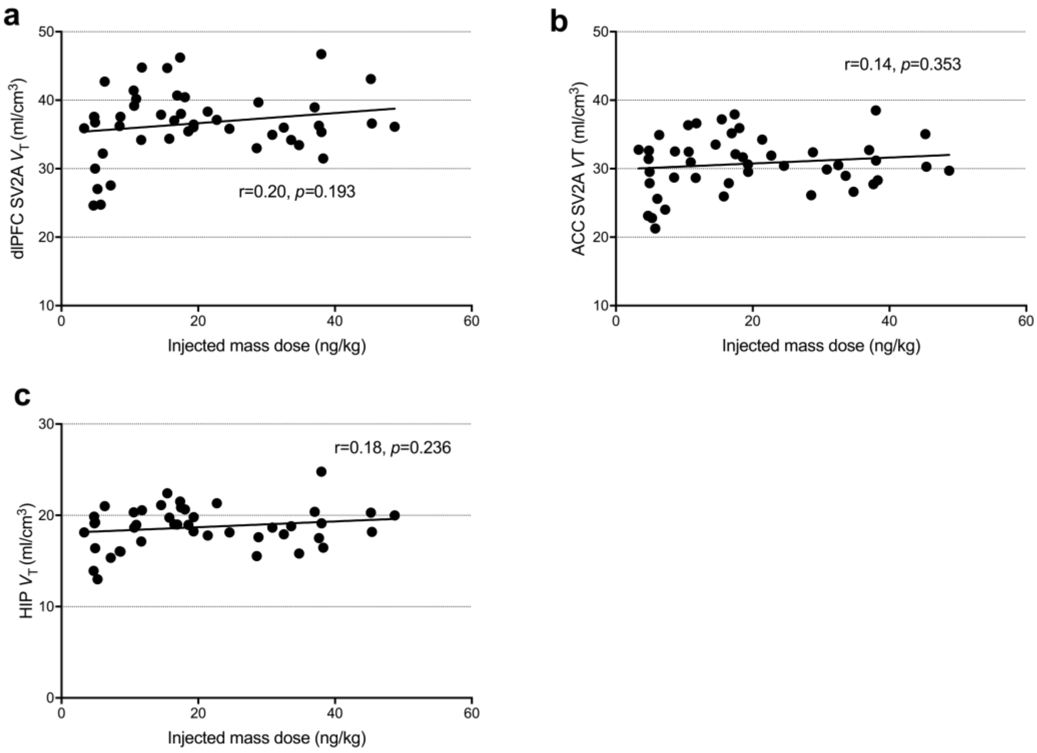

Supplementary Figure 2. Correlation between extracted dlPFC-PCC connectivity values and dlPFC synaptic density

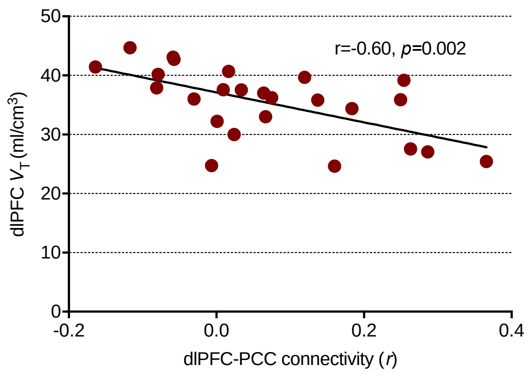

Supplementary Figure 3. Centrum semiovale V_T across groups

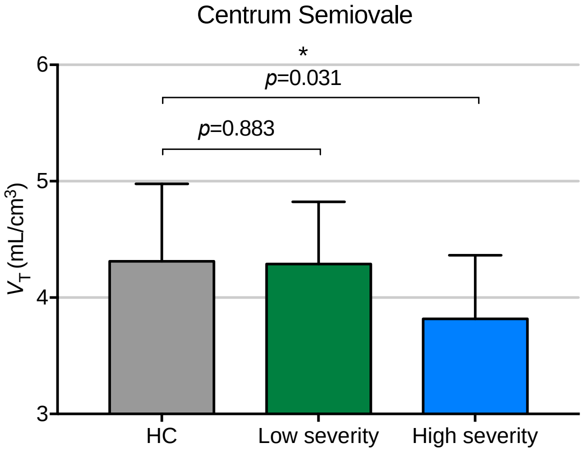

Supplementary Figure 4. Single group dlPFC seed connectivity results

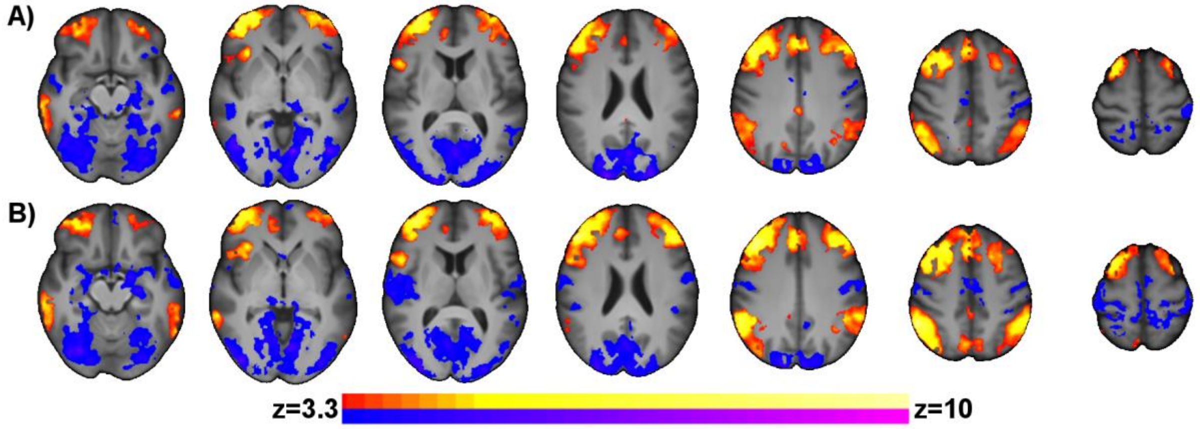

A.Transverse slices of connectivity maps from dlPFC seed in HCs. B. dlPFC seed connectivity maps for clinical subjects.

Supplementary Notes

V_T/f_p was significantly negatively correlated with depressive symptoms (HAMD-17) across all clinical subjects in primary ROIs: dlPFC (r=-0.613, *p*=0.001), ACC (r=-0.638, *p*<0.001), hippocampus (r=-0517, *p*=0.007). All correlations survived corrections for multiple comparisons.

Correlations between V_T and depressive symptoms were significant without PVC correction in primary ROIs. dlPFC: r=-0.501, *p*=0.009; ACC: r=-0.504, *p*=0.009; hippocampus : r=-0.420, *p*=0.033.

There were no main effects of sex on V_T across groups in primary ROIs - HC: $F_{3,17}$=0.528, *p*=0.669; low severity: $F_{3,10}$=0.218, *p*=0.882; high severity: $F_{3,8}$=0.233, *p*=0.871. There were no main effects of smoking on V_T across groups – HC: $F_{3,17}$=1.477, *p*=0.256; low severity: $F_{3,10}$=1.945, *p*=0.186; high severity: $F_{3,8}$=0.138, *p*=0.935.

There were no significant correlations between V_T and PTSD symptoms (PCL-S) in the individuals with PTSD (n=13); dlPFC: r=-0.023, *p*=0.941; ACC: r=-0.023, *p*=0.941; hippocampus: r=0.201, *p*=0.510.

Supplementary Methods

[^{11}C]UCB-J was synthesized as reported previously (Nabulsi et al. 2016). ^{11}C-methyl iodide was swept through a well degassed and acetone/ice bath cooled solution of 8:1 DMF/water (v/v, 250 µL) containing 0.3-0.32 mg of tris(dibenzylideneacetone)-dipalladium(0) ($Pd_2(dba)_3$), 0.45-0.5 mg of tri(*o*-tolyl)-phosphine (P(*o*-tol)$_3$), and 1 mg potassium carbonate. After radioactivity peaked, 1-1.2 mg the precursor (see note 1) in 100 µL of 8:1 degassed DMF/water was added, then the reaction vial was placed in a heating block preheated at 100 °C, and was allowed to stir for 5 min at this temperature. The reaction solution was cooled in an acetone/ice bath, then diluted with 1.6 mL of 1 N HCl, filtered through a 0.45 µm membrane filter (13 mm Millex-HV PVDF) and loaded onto a Gemini-NX C18 preparative HPLC column (5 µm, 10 × 250 mm) for purification. The column was eluted with acetonitrile/0.1 M ammonium formate /saturated ammonium hydroxide (35:65:1.3, v/v, aq. phase pH ~10) at a flow rate of 2 mL/min for the first 3 min, and 5 mL/min thereafter. The desired radioactive product fraction was collected (t_R = ~18 min), diluted with 50 mL of de-ionized (DI) water, and passed through a Waters C18 Classic SepPak cartridge. The SepPak cartridge was rinsed with 10 mL of 1 mM HCl. The radioactive product was recovered by eluting the SepPak with 1 mL of absolute ethanol (USP), followed by 3 mL of saline (USP). This mixture was then passed through a sterile membrane filter (25 mm, 0.22 µm Millex-MP) for terminal sterilization and collected into a sterile vial precharged with 7 mL of saline (sterile, USP) and 40 µL of 4.2% sodium bicarbonate (sterile, USP) affording a formulated solution ready for dispensing and administration. The radiochemical purity and molar activity were determined by HPLC analysis of the final product solution (column: Genesis C18, 4.6 × 250 mm, 5 µm; mobile phase: 38% acetonitrile and 62% 0.1 M aqueous ammonium formate with 0.5% acetic acid (v/v, pH 4.2); flow rate: 2 mL/min; ultraviolet detector wavelength: 261 nm). The identity of [^{11}C]UCB-J was confirmed by coinjection of the product with the unlabeled UCB-J. The average yield at end of synthesis (decay uncorrected) was 10.9 ± 3.6% (n=78), with average molar activity of 54.7 ± 27.4 mCi/nmol at end of beam, and average radiochemical purity of 99.6 ± 0.2%. The average radiosynthesis time was 56 ± 3 minutes. Note: The trifluoroborate precursor contained 3% of the boronic acid derivative.

Supplementary Figure 5. Synthesis of [^{11}C]UCB-J

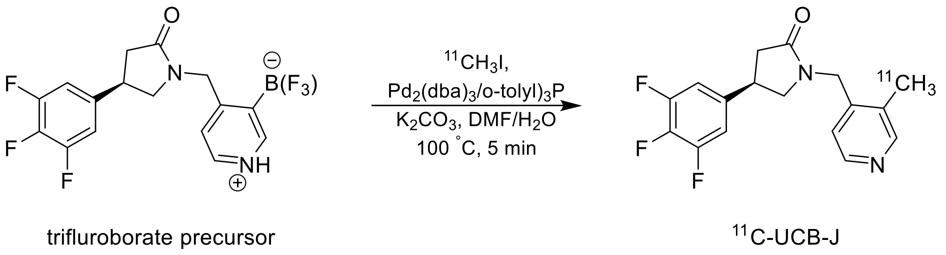

Before [^{11}C]UCB-J injection, a 6 minute transmission scan was performed for attenuation correction. PET data were acquired in list-mode for 120 min after intravenous infusion of [^{11}C]UCB-J over 1 min by an automated infusion pump (Harvard PHD 22/2000; Harvard Apparatus, Holliston, MA, USA). The dynamic emission data were reconstructed into 33 frames (6 x 0.5min, 3 x 1min, 2 x 2min, and 22 x 5 min) with corrections for attenuation, normalization, scatter, randoms, and dead time using the MOLAR algorithm. Event-by-event motion correction was included in the reconstruction based on motion detection with a Polaris Vicra optical tracking system (NDI Systems, Waterloo, Canada).

All subjects underwent arterial cannulation and blood was collected for measurement of the time course of [^{11}C]UCB-J in plasma, including radiometabolite analysis. Samples were drawn every 10 s for the first 90 s and at 1.75, 2, 2.25, 2.5, 2.75, 3, 4, 5, 6, 8, 10, 15, 20, 25, 30, 45, 60, 75,

90, 105, and 120min after [^{11}C]UCB-J injection. Radiometabolite analyses were performed for plasma samples at 3, 8, 15, 30, 60, and 90min using an automatic column-switching HPLC system. The unmetabolized parent fraction was determined as the ratio of the radioactivity corresponding to the parent to the total amount of radioactivity collected and fitted with an inverted integrated gamma function. The curve was normalized with the time-varying extraction efficiency, which was determined by corresponding reference plasma samples and fitted with an exponential function. The arterial plasma input function was calculated as the product of the total plasma activity, the [^{11}C]UCB-J HPLC fraction curve, and the extraction efficiency curve. Plasma free fraction (f_p) was measured in triplicate using an ultrafiltration method (Millipore Centrifree micropartition device 4104, Billerica, MA, USA). The f_p values were determined as the radioactivity ratio of ultrafiltrate to plasma.
